# Kirkes' Hand-Book of Physiology

**Published:** 1897-03

**Authors:** 


**Kirkes' Hand-Book of Physiology.** By W. D. Halliburton, M.D., F.R.S. Fourteenth Edition. Pp. xvii., 851. London: John Murray. 1896.

Kirkes' well-known Hand-book has, in its fourteenth edition, been to a great extent rewritten by Professor Halliburton: it retains its original form, and has many of the old familiar illustrations; but a large number of the six hundred drawings are new and are a useful addition to the volume, which, excepting in some minor details, is essentially a new one. For many years the older editions have supplied the wants of the medical student, giving him a combination of histology with physiology proper; this arrangement has been preserved, and we think rightly so: other text-books on physiology give a greater amount of detail, but here we have just the combination which the average student requires. Professor Halliburton's name is a guarantee that the work is done by one who knows, and he has avoided the common mistake of giving too much space and attention to any one branch of the subject. His account of the structure, properties, and mode of coagulation of the blood leaves little to be desired; but we should like to see somewhat more notice taken of the clinical methods of obtaining the specific gravity of blood, inasmuch as we have found that Roy's method, modified by Lloyd Jones, is a more trustworthy guide as to the quality of the blood from the clinical standpoint than is the Hæmoglobinometer, which gives such varying and untrustworthy results in the hands of those who are not very sensitive to delicate shades of colour.

The chemistry of the action of the gastric juice is briefly described, and the various intermediate varieties of proteoses are indicated: some text-books leave the student in absolute chaos over the different modifications of proteids, but no one need be in any dilemma after perusing this book.

We are glad that the author has utilised the latest methods for an analysis of the distribution of nerve fibres, inasmuch as no book on histology should ignore the method of Golgi; the author's analysis of the structure and working of nerve tissues is very clear and concise.

We congratulate him on having kept the volume within reasonable limits of size without the sacrifice of any important details.

